# Smart haptic gloves for virtual reality surgery simulation: a pilot study on external ventricular drain training

**DOI:** 10.3389/frobt.2023.1273631

**Published:** 2024-01-10

**Authors:** Jonah Boutin, Jafer Kamoonpuri, Reza Faieghi, Joon Chung, Sandrine de Ribaupierre, Roy Eagleson

**Affiliations:** ^1^ Schulich School of Medicine and Dentistry, University of Western Ontario, London, ON, Canada; ^2^ Department of Aerospace Engineering, Toronto Metropolitan University, Toronto, ON, Canada; ^3^ Department of Electrical and Computer Engineering, University of Western Ontario, London, ON, Canada

**Keywords:** virtual reality, haptics, haptic feedback, surgery simulation, surgical education

## Abstract

Smart haptic gloves are a new technology emerging in Virtual Reality (VR) with a promise to enhance sensory feedback in VR. This paper presents one of the first attempts to explore its application to surgical training for neurosurgery trainees using VR-based surgery simulators. We develop and evaluate a surgical simulator for External Ventricular Drain Placement (EVD), a common procedure in the field of neurosurgery. Haptic gloves are used in combination with a VR environment to augment the experience of burr hole placement, and flexible catheter manipulation. The simulator was integrated into the training curriculum at the 2022 Canadian Neurosurgery Rookie Bootcamp. Thirty neurosurgery residents used the simulator where objective performance metrics and subjective experience scores were acquired. We provide the details of the simulator development, as well as the user study results and draw conclusions on the benefits added by the haptic gloves and future directions.

## Introduction

Virtual Reality (VR) in surgery simulation is still an underdeveloped research field, though it has the potential to become an effective training tool for surgeons. Traditional apprenticeship is still the standard practice in many surgical training programs, and the well-known rule of 10,000 h of practice to master a skill is the ideal target for surgical training programs (Anders Ericsson, 2008). However, in traditional apprenticeship training, achieving a high volume of practice is challenging, primarily due to the limited availability of cadavers and animals, excessive cost, safety risks, and the emphasis on patient safety and work restrictions. VR surgical simulation is an emerging technology that is presently believed to address this challenge in surgical training. Not only do these simulations eliminate the stringent need for patients or cadavers, but they also provide structured feedback on the surgeons’ performance by quantitatively assessing the completed operation (Armstrong et al., 2016). In fact, a meta-analysis of 14 studies revealed that simulation-based training was a more effective training method than the traditional apprenticeship (McGaghie et al., 2011).

The addition of realistic haptic feedback in VR surgery simulation is an ongoing area of research; see (Faieghi et al., 2020a) and references therein. Although existing studies have shown that using inanimate surgical simulators results in the acquisition of surgical skills, further research is required to facilitate quantifying surgeons’ skills on these tools (Keyser et al., 2000; Satava, 2001; Fraser et al., 2003; Eagleson and Joskowicz, 2023). Computerized simulators record direct tracking of individual performances which can be carefully assessed to provide constructive feedback to the trainee (Duffy et al., 2004). However, many surgical simulators solely rely on VR environments without incorporating haptic feedback (Latham et al., 2019), resulting in a low immersion in the simulation environment, which in turn results in quantitative feedback that is not an accurate representation of the surgeon’s capabilities. Even though many papers have investigated solutions to these challenges, there are very few that provide a simulated neurosurgical environment that includes haptic feedback and an effective scoring method.

Given the growing attention to smart gloves and their potential to improve immersion in VR, we conducted a study to explore the usability and effectiveness of haptic gloves among surgical residents. To our knowledge, there is no other study that has explored the application of smart haptic gloves in the context of surgery simulation and surgical training. After consulting with expert neurosurgeons, it was decided that an ideal operation to simulate was external ventricular drain placement.

The placement of External Ventricular Drain (EVD) is one of the most performed neurosurgical procedures, often performed at the bedside by junior residents, and consequently, is an essential skill to be mastered by neurosurgical trainees early in their careers (Ghandorh et al., 2017). An EVD is a flexible plastic catheter used to treat hydrocephalus and relieve elevated intracranial pressure when the normal flow of cerebrospinal fluid inside the brain is obstructed. The optimal placement of the drain involves choosing an appropriate burr hole on the skull and blindly placing a catheter through the burr hole to intersect a lateral ventricle to drain cerebrospinal fluid and relieve intracranial pressure. Undesirable trajectories lead to multiple tries to hit the ventricle, with the potential risk of damaging eloquent brain areas (Rossitto et al., 2023). For further discussions on the challenges of this operation and current trends in training for this operation see (Perin et al., 2018; Yuen et al., 2018; Austerman et al., 2020).

The proposed simulator for this operation will allow residents to acquire these targeting skills before attempting the placement on live patients, reducing the risk of operations (Kakarla et al., 2008; Camacho et al., 2011). Success can be easily quantified using the trajectory of both the drill and the drain placement, which makes it the ideal procedure to perform in a virtual environment.

Based on the details mentioned above, this paper will demonstrate the use of a haptics-enabled VR simulator for an EVD placement. The simulator was made using commonly available open-source software tools Unreal Engine^1^ and Blender^2^ programs and was combined with haptic gloves and a head-mounted display for the user to view and interact with the virtual environment. This surgical training tool will use a combination of haptic feedback and performance assessment metrics to assess a surgeon’s ability to perform this operation, providing quantitative feedback on the surgeon’s performance which can become very useful in training.

Over the past years, haptic gloves evolved for hand-based human-computer interaction. Recent advancements offer real-time hand and finger pose tracking, alongside vibrotactile feedback, enhancing VR experiences. Although kinesthetic haptic devices in surgery simulation are well-researched, there is a lack of studies on haptic gloves for feedback delivery.

## Methods—system design

### Software/hardware overview

The most important component of the simulator for our study was the SenseGlove NOVA^3^ haptic gloves. The two major components of the gloves are the “hub” and the “soft glove.” The soft glove provides comfort to the user, while the hub contains a vibrotactile actuator, responsible for creating vibrations along the back of the hand, and a Linear Resonant Actuator (LRA) located on the tip of the thumb and index finger. A VIVE tracker^4^ is mounted to each glove, to track the position of the hand in 3D space. Steam VR^5^ was used for proper tracking of both VIVE trackers on the gloves as well as the VR headset, and the gloves were connected to the environment using SenseCom, an application created by SenseGlove. Further documentation on the software/hardware components of the haptic gloves can be accessed publicly through SenseGlove^6^. This setup was combined with a VR head-mounted display to immerse the user in a virtual environment created using Unreal Engine 4.27.

### Graphical environment

Since eye-hand coordination and taking an accurate trajectory of the surgery tool is critical for the EVD operation, there were technical challenges in simulating this operation. The 3D models needed to be accurate, and the collision detection algorithms of each surgical tool in the environment needed to be as realistic as possible to accurately simulate the operation. Models were acquired from TurboSquid^7^ and then imported into Blender for further changes. These modifications included applying mesh repair algorithms to fix topological and geometrical errors, as well as scaling and refining mesh resolution to meet the visual and computational requirements of the simulator. All the collision detection algorithms between objects were programmed directly in Unreal Engine’s platform, and this was what provided the ability to simulate a burr hole in the patient’s skull. The simple bounding box collision methods available in Unreal proved to be sufficient in providing a reasonably accurate representation of the burr hole. As for the rest of the objects in the environment, they were all modified using a combination of Blender and GIMP to provide accurate models to produce a realistic simulation.

### Haptic environment

A haptic glove application program interface is already integrated into the Unreal Engine platform which allows the user to manipulate objects within the Unreal VR environment using haptic gloves. In addition, there is also an ability to command arbitrarily vibrotactile feedback to different joints of the haptic glove. The haptic glove chosen for this simulator was the SenseGlove NOVA, which are wireless force-feedback gloves compatible with standalone headsets that provide comfort and functionality to the trainee. Initially, the coordinates of the haptic gloves were registered in the coordinate system of the graphical environment, which allowed for the ability to compute appropriate haptic feedback to be delivered to the user’s hand wearing the glove. Since the gloves function based on creating a resistive force on each finger and increasing the force proportionally to the amount of finger curl detected, the level of force was adjusted based on the material properties of the object in the user’s hand. The level of force was adjusted to all materials in the environment including the surgical drill, the catheter, and the patient’s skull. The surgical drill required further adjustments, with various levels of vibrotactile feedback assigned to each material the drill encountered. The intensity of the feedback oscillated as the drill passed through tissue and air before reaching the skull, at which point its intensity reached a maximum until the skull had been fully penetrated. After penetration, the intensity dropped to a minimum indicating that the surgeon should immediately stop the drill. The specific values for the change in vibration were assigned through a trial-and-error process with expert neurosurgeons. The user feels an initial vibration once the drill trigger is pressed, and that vibration is multiplied by a factor of 3 when the drill makes contact with the tissue, and a factor of 5 once the drill has made contact with the skull.

To further improve realism, we also generated resistive force for the fingers that come into contact with the virtual drill button. The intensity of this force is set proportional to the level of finger curling. We controlled the rotational speed of the drill based on the amount of force applied to the drill button. This led to an improved realism in simulating interactions with the drill and adjusting its rotational speed using the drill button.

Once all vibrotactile feedback and force levels were adjusted to all objects in the environment, the haptic feedback was demonstrated to an expert neurosurgeon to assess realism. After consultation with the surgeon, the feedback magnitudes were adjusted to reach a level of realism that provided for an accurate surgical simulation.

## Methods—experimental setup

### Design of task

**FIGURE 1:**
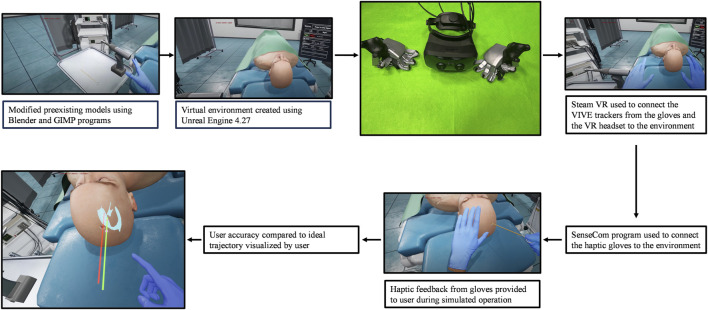
Overview of the proposed simulator’s components with images provided.

**FIGURE 2:**
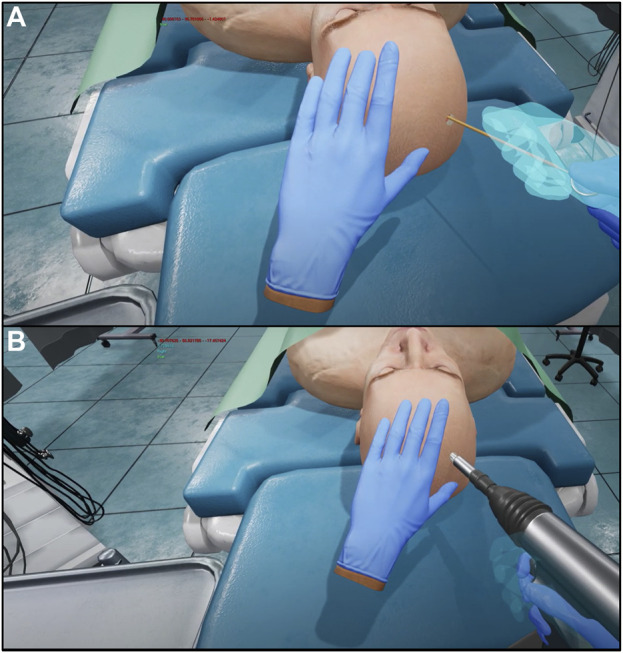
Screen capture of ghost hand (shown in transparent blue), compared to virtual hand (shown wearing blue glove) during catheter insertion **(A)**. Screen capture of ghost hand (shown in transparent blue), compared to virtual hand (shown wearing blue glove) during creation of the burr hole **(B)**.

The surgical operation implemented in the virtual environment was selected for its importance in neurosurgical education. The external ventricular drain placement procedure is a routine neurosurgical operation that is one of the most common and important lifesaving procedures in the neurologic intensive care unit (Muralidharan, 2015). Ventricular models imported had been used previously in our lab and had been designed to provide a variety of difficulties, all clinically realistic for different pathologies and scenarios (Armstrong et al., 2014; Armstrong et al., 2016). Due to the positive effects of this procedure such as draining blood and cerebrospinal fluid to mitigate intracranial hypertension (Muralidharan, 2015), this is an ideal operation to provide a new training method for. Once the user has been set up with the haptic gloves and VR headset as seen in the flow chart described in Figure 1, they are immersed in a virtual operating environment. They are then directed to type their name on a virtual keyboard, using the SenseGlove NOVA’s haptic features. Once the users have entered their names, they then select which side of the patient’s head they will operate on first. Once this information has been filled out, users may select the start button and close the menu. At this time, the user may begin the procedure. This simulation contains the most important parts of the procedure, in consultation with expert neurosurgeons. First, the trainee will use the virtual drill to place burr holes in the correct location on the patient’s head, feeling a vibration in the gloves as well as changes in pressure once the skull has been penetrated. Throughout the whole procedure, the trainee can visualize the “ghost hand” feature shown in Figure 2. This feature allows the surgeon to align their hand (blue transparent hand) with the VR hand (gloved hand) that shows them the speed at which they should be moving forward to safely create the burr hole. The trainee will then insert a virtual catheter into the burr hole, sensing pressure changes upon penetration. After achieving the desired depth, the attempt is ended by pressing the stop button in the environment. Users can review their catheter placement and accuracy compared to experts in Figure 1. They can then start a new attempt on the opposite side.

### Performance metrics

It is important to be able to train both speed and accuracy for this procedure since both factors are important in an emergent situation in a clinical setting (Ofoma et al., 2022). For this reason, we used the following scoring method which accounts for both speed and accuracy. The development of this metric is fully explained in our recent study (Eagleson et al., 2022). The exact score is calculated using Eq. (1), and a brief explanation is provided here.

$$Score=N—\left(t_{h}∙d_{h}\right)-\left(t_{c}∙d_{c}\right),$$ (1)

where N is an arbitrary large number, $t_{h}$ represents the time from the start of the procedure to when the burr hole was made (in milliseconds), $d_{h}$ represents the magnitude of a vector between the location where the burr hole was made and the ideal burr hole location (in centimetres), $t_{c}$ represents the time from the start of the procedure to when the catheter punctured the ventricle (in milliseconds), and $d_{c}$ represents the magnitude of a vector from the location where the ventricle was punctured and the ideal puncture location (in centimeters). The value of N is chosen such that the number of digits fits well in the aesthetics of the information panel in the simulator ( N=999999 in this case). While this equation seems simple, it was deemed most effective after deliberations with expert neurosurgery surgeons. When using this equation, the trainee needs to get the lowest value possible in all measured values in order to have the lowest impact on the N value. This method ensures that the trainee focuses on accuracy as well as speed. This is also a very important factor as this procedure is frequently an emergent, lifesaving procedure (Chung et al., 2019). The comparison between ideal placement and actual placement can be visualized by the user, as shown in Figure 1. When using this formula, the best score is the one with the highest number, because this shows that the trainee used a combination of both speed and accuracy to complete the procedure.

### Experimental procedure

**FIGURE 3:**
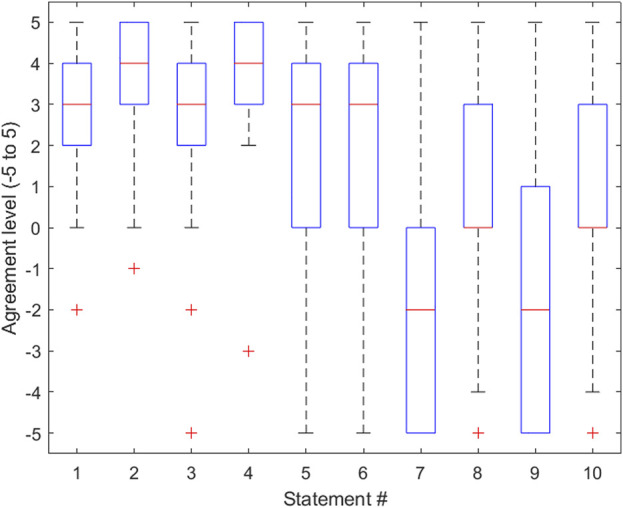
Boxplot representing the distribution of data gathered for each statement in the survey provided in Table 1. The lower half of each box represents the first quartile of the data, the middle of the box represents the second quartile of the data, and the top half of each box represents the third quartile of the data. The whiskers of the boxplot represent the minimum and maximum data points and their variability in comparison to the interquartile range. Outliers are shown. This boxplot was created using MATLAB software.

The simulator underwent testing at the 2022 Canadian Neurosurgery Rookie Camp in London, Ontario, where all new neurosurgery residents from across Canada gathered for 3 days of training. Thirty beginner residents, unfamiliar with haptic gloves and VR, received the same simulator instruction and setup. Once set up, the subjects were each given 3 min on the simulator to complete as many attempts as possible, following the exact operation outlined in the *Design of task*. Each subject’s score was recorded, and the best score out of the 30 subjects was announced at the end of the testing period to incentivize each resident surgeon to give it their best effort. Subjects were asked to complete a survey once they had completed their attempts. This survey is presented in Table 1, and the data is further analyzed in Figure 3. The survey’s main goal was to gain feedback to inform design specifications for future iterations of the simulator. To accomplish this, the survey consisted of questions that focused on assessing the potential of the simulator as a training device, the influence of the haptics involved within the simulator, and the accuracy of the simulator in terms of how well it was able to represent the surgeon’s capabilities, as will be discussed in the next sections.

**TABLE 1:** Survey questions and responses.

Statement	Agreement score (−5 to 5)	
	Average	Variance
I feel that this particular VR training tool can provide a reasonable assessment of clinical skills performance.	2.6	3.3
Computer-based technical skill training systems will represent a cost-effective form of instruction.	3.8	3.2
I think that haptic gloves have potential to reasonably simulate a surgical drill.	2.4	6.4
The headset and haptic gloves were comfortable and did not hinder my ability to complete the procedure.	3.7	2.6
Surgical trainees should be trained on these simulators prior to performing the procedure for the first time.	2.0	7.79
I feel that using this simulation on a regular basis would greatly enhance my ability to perform this procedure.	2.1	7.99
Frankly, the experimental session’s impression of pessimism and uncertainty caused me to lower my rankings.	−2.2	9.96
Frankly, the experimental session’s impression of enthusiasm and optimism caused me to raise my rankings.	0.61	9.77
Frankly, I felt obliged/rushed to run in this data-gathering game-like study and that lowered my performance.	−1.8	10.72
Frankly, my awareness of running in an experimental data-gathering game-like study raised my performance.	0.39	7.27

## System design results/discussion

The survey consisted of 10 statements from which the residents rated their agreement with the statement on a scale that ranged from −5 to 5, where −5 represented the greatest level of disagreement and 5 represented the greatest level of agreement. For the purpose of this paper, we will refer to this value as the “agreement score.” When given the statement “I think that haptic gloves have the potential to reasonably simulate a surgical drill,” residents responded with an average agreement score of 2.4. This was an important indication of the added value of haptic gloves in improving the surgery simulation experience. This data also provided the conclusion that the method used to simulate the surgical drill was indeed effective. Another important piece of information gathered from the survey was from the statement “The headset and haptic gloves were comfortable and did not hinder my ability to complete the procedure.” The average agreement score used to respond to this statement was 3.7. This score implies that the equipment described in the *Software/hardware overview* section of this paper was not only effective in replicating the surgical procedure as concluded earlier but also effective in providing the user with a comfortable experience, in keeping with the simulator’s goal of providing a score that accurately reflects the user’s abilities. Importantly, several residents commented that the simulator lacked the ability for the surgeon to use one hand to stabilize themselves on the patient’s skull prior to inserting the catheter. This can be considered in future iterations of the simulator to elevate the simulation realism. Another potential feature of the simulator that can be investigated is the possibility of calibrating the virtual hand size with the trainee’s hand size, with the intention of adding an extra level of realism to the virtual environment. Since the survey respondents were all neurosurgical residents, their feedback on the accuracy and comfort of the overall environment can be considered when improving the simulator. Ideally, the current smart gloves should be updated in a way that allows us to better manipulate the haptic feedback received by participants, using kinesthetic haptic rendering methods (Faieghi et al., 2020a). Additionally, the ability to measure this haptic feedback quantitatively would be an incredible asset to trainees as it would provide instructors with a method of assessing the power used by the trainee as they puncture the skull and ventricle. The most important next step gathered from this portion of the results is the necessity of a feature that allows the user to stabilize themselves while attempting to insert the catheter into the ventricle.

## Experimental results/discussion

Results were gathered from the demonstration performed at the 2022 Canadian Neurosurgery Rookie Camp, via survey responses gathered once the surgical residents had completed their attempts. Survey statements consisted of a range of questions including those that assessed the resident’s opinion on the realistic level of the simulator as well as the effectiveness of the equipment described in the *System design* portion of the *Methods*.

When given the statement “I feel that this particular VR training tool can provide a reasonable assessment of clinical skills performance,” the average agreement score given by residents was 2.6. This was a critical statement with respect to assessing the realism of the simulator. With a score of 2.6, it is reasonable to conclude that in the eyes of the residents training on the simulator, it is a valid tool for surgical education.

When given the statement “Surgical Trainees should be trained on these simulators prior to performing the procedure for the first time,” the average agreement score given by residents was 2.0. This statement reflects the fact that not all surgical residents who train on the simulator will have prior experience with virtual environments and haptic gloves. Due to this, the user may experience difficulties in their first few attempts, which could potentially affect their scores. A solution to this issue that could be explored in the future is implementing an instructional portion of the simulator, that demonstrates to the user how to operate and interact with objects while in the virtual environment.

Another important statement that was provided to users was “I feel that using this simulation on a regular basis would greatly enhance my ability to perform this procedure.” The average agreement score given in response to this statement was 2.1. This statement was effective in gathering an idea of the necessity of this simulator. The group of participants answering this survey was comprised of 30 surgical residents from across Canada, so a response of 2.1 to this statement signifies that these particular residents feel that the simulator would be a useful tool in their surgical education.

Another statement that should be noted was “Frankly, I felt obliged/rushed to run in this data-gathering game-like study and that lowered my performance.” The average agreement score given when responding to this statement was −1.8, representing more of a disagreement with the statement than an agreement. This response is invaluable, because it shows that the participants felt they were performing naturally while they were immersed in the virtual surgical environment. As such, one can speculate that scores gathered from the simulator can be considered relevant with regard to representing the resident’s surgical abilities.

Additionally, certain residents mentioned that they would have felt more comfortable performing their first EVD Placement if they had had the chance to utilize this training device beforehand. They indicated that using the virtual tool could provide a higher level of confidence before their first operation.

One limitation of the current design was concluded based on feedback from several residents that mentioned they have used their own hands for landmarking. As the size and shape of the hand in the simulator do not necessarily match the user’s hand, this can potentially limit the usefulness of the simulator. Therefore, the addition of a feature that can change virtual hand size according to the user’s hand size and shape will improve the practical relevance of the simulator.

The variance for all the statements was calculated and is shown in Table 1. In statements 1, 2, and 4 it should be noted that the variances were insignificant (all below 3.3), and their average agreement scores were also large, 2.6, 3.8, and 3.7, respectively. This was an important finding, because these three statements assessed the simulator’s ability to comfortably and accurately assess the surgeon’s performance, and the relationship shown between the average and the variance agreement score proves that the trainees who responded to the survey all responded to these statements with a value close to the mean, which was high.

The results of this survey provided meaningful conclusions about the simulator, as well as insight into the future modifications that can be made. Most notably, calculated agreement score averages to statements provided conclusions that the simulator is accurate in assessing surgical ability and would provide a useful surgical training tool, with respect to the sample group of 30 neurosurgical residents that attended the 2022 Canadian Neurosurgery Rookie Camp.

## Conclusion

Our user study survey validated aspects of the simulator’s accuracy and suggested future improvements. Participants’ answer to the statement about the VR tool’s assessment capability affirmed its accuracy in evaluating clinical skills. Users emphasized the need for stabilization during the procedure, highlighting an important improvement area. This pioneering exploration of haptic gloves in surgical training requires more extensive data collection for training progress assessment. While the user study shows promise, further research is necessary to refine this technology.

It is worth noting the limitations of this study. While we used one of the latest commercially available smart haptic gloves in this work, the glove was only able to generate vibrotactile feedback, resulting in a lack of kinesthetic feedback. Addressing this will require the design of new mechanisms that can provide both vibrotactile and kinesthetic feedback to the user. Moreover, it appears that the current state-of-art smart haptic gloves have limitations in certain aspects of the vibrotactile feedback that affected the accuracy of the simulator. Although the design team and expert neurosurgeon involved in the development of the simulator unanimously confirmed that the generated vibrotactile feedback had a positive impact on the simulation experience, future haptic glove simulators will benefit from more customization capabilities so that advanced haptic rendering algorithms such as the ones with varying frequency (Faieghi et al., 2020b) can be implemented to better replicate material properties. Furthermore, there were certain limitations in the experiment conducted. Future studies should consider the addition of a control group that does not use haptics in their surgical simulation, with the goal of presenting a better view on the advantages provided by haptic interaction.

Future enhancements for this simulator will address issues, potentially through new hardware designs, and including instructional methods for user comfort and accuracy. Overall, the simulator realistically replicates External Ventricular Drain Placement, bridging haptics and surgical simulation. It is a pioneering use of haptic gloves in surgical simulation, with the potential for significant progress in the field. However, further research and a comprehensive study are needed to establish its effectiveness in improving surgical skills.

## Data Availability

The raw data supporting the conclusion of this article will be made available by the authors, without undue reservation.

## Footnotes

1
https://www.unrealengine.com

2
https://www.blender.org

3
https://www.senseglove.com/product/nova

4
https://www.vive.com/ca/accessory/tracker3

5
https://store.steampowered.com/steamvr

6
https://senseglove.gitlab.io/SenseGloveDocs/nova-glove.html

7
https://www.turbosquid.com/
